# Examining the American mindset on community well-being: insights from a national survey

**DOI:** 10.1186/s12889-026-26507-0

**Published:** 2026-02-11

**Authors:** Linnea Warren May, Adaeze Ibeanu, Delia Bugliari, Ruolin Lu, Anita Chandra

**Affiliations:** 1 RAND, 4570 Fifth Ave #600, Pittsburgh, PA 15213 USA; 2 RAND, 1776 Main Street, Santa Monica, CA 90401 USA; 3 RAND, 1200 S Hayes St, Arlington, VA 22202 USA

**Keywords:** Community well-being, Community conditions, Well-being measurement, Health mindsets, Thriving

## Abstract

**Background:**

The United States is experiencing a crisis of well-being, characterized by increasing social isolation, hopelessness, and diseases of despair. Traditional well-being measures have focused on individuals, often overlooking community dimensions that are critical for understanding and addressing broader societal challenges. This study primarily aims to identify the factors most strongly associated with perceptions of community well-being.

**Methods:**

We analyzed data from the nationally representative 2023 National Survey of Health Attitudes (NSHA; *N* = 5,620). Logistic regression models were used to examine associations between well-being measures and demographic characteristics and factors related to community conditions and connections.

**Results:**

Perceptions of overall community well-being varied by income, age, education, and race/ethnicity. Individual well-being was not significantly associated with perceptions of community well-being. Among community conditions, access to outdoor spaces (OR = 2.50), healthy food (OR = 1.67), and health care (OR = 1.39), and safe drinking water (OR = 1.24) were significantly linked to higher community well-being ratings, while transportation infrastructure was negatively associated (OR = 0.77). For community connections, trust among members (OR = 2.08), mutual support (OR = 1.74), and collaboration for health (OR = 1.32) also showed significant associations. These results suggest the importance of both physical resources and social infrastructure for community well-being.

**Conclusion:**

Multidimensional measures of both individual and community well-being can help local leaders and policymakers monitor well-being, set priorities, and design interventions.

**Supplementary Information:**

The online version contains supplementary material available at 10.1186/s12889-026-26507-0.

## Background

There is growing recognition that the United States faces a complex crisis of well-being, characterized by rising social isolation, hopelessness, and diseases of despair, happening even as healthcare spending has increased [1, 2]. Many Americans report feeling disconnected from each other and uncertain about the future, and national surveys highlight a widespread belief that life has become more challenging in recent years [3, 4]. These concerns extend beyond individual dissatisfaction, as hopelessness and disconnection are linked to broader mental health and economic challenges that affect families and communities alike [5, 6]. In response, communities across the country are increasingly seeking to understand the underlying factors that shape well-being and are exploring practical, accessible ways to measure how their members are faring—not just as individuals, but collectively as part of a community.

Well-being can be understood at multiple levels—individual, community, and collective [7] —yet most measurement efforts to date have focused primarily on individual well-being, with less attention to community or collective dimensions. Perceptions of both personal and community well-being can vary widely across different demographic groups and contexts. For instance, Black Americans often report higher psychosocial well-being but lower financial security compared to White Americans, perhaps reflecting the buffering effects of strong social and cultural support networks alongside persistent economic disparities [8], while Asian Americans tend to report high well-being across most domains, potentially due to stronger economic stability and health indicators on average [9, 10]. Historically, well-being has shown a U-shaped pattern across the lifespan, tending to dip in midlife before rising again in later years. Research now points to declines during young adulthood, which may reflect the unique economic and social pressures experienced in early adulthood, thereby altering the classic trajectory and suggesting a more complex age–well-being relationship [11–13]. Income and education are generally predictors of higher individual well-being, though their effects differ by gender and race and often interact in complex ways. For example, the returns to education and income for well-being are not uniform across groups, as social and structural factors—including perceived inequity, discrimination, and access to opportunity—can moderate these relationships [14–16]. Consequently, racial differences in reported well-being cannot be explained solely by objective circumstances such as income or education but also reflect how individuals perceive fairness, inclusion, and social standing when evaluating their overall satisfaction and quality of life [14, 15]. Additionally, recent data also show that Americans are generally more optimistic about their personal futures than about the future of their communities or the nation [17, 18]. Taken together, these findings underscore the importance of measuring well-being at multiple levels and understanding whose perspectives are represented in those measures.

Health mindsets—people’s beliefs and assumptions about the causes and responsibilities for health—play a critical role in shaping expectations, behaviors, and support for policies that promote health and well-being [19–21]. For example, shifting individual and collective mindsets toward viewing health as a shared value, rather than a personal privilege, is essential for fostering equitable opportunities and driving social change to improve health outcomes [19]. The National Survey of Health Attitudes (NSHA) has provided valuable insights into American health mindsets since 2015, and recent waves of the survey have captured information to understand how people think about different aspects of well-being, including perceptions of individual and community well-being [22]. Understanding mindsets around well-being can inform the design and sustainability of interventions, foster community advocacy, and strengthen collective efforts to promote health and well-being [19]. 

Additionally, simple, accessible tools that assess both individual and community well-being can support more comprehensive understanding of how personal experiences relate to local conditions, helping communities track trends and identify areas for improvement. Broadening approaches to well-being measurement can in turn guide policies and programs toward factors such as social connection, inclusion, and equitable access to resources that are known to influence well-being at multiple levels [23, 24]. 

### Concepts of individual and community well-being

Individual *well-being* is a multidimensional concept that encompasses both objective and subjective aspects of life, and is influenced by, but more expansive than *health* [25]. It is often defined as the degree to which people experience their lives as favorable and aligned with their needs and goals [26]. Objective indicators, such as income, education, and physical health, reflect material and structural conditions that support quality of life, while subjective indicators capture people’s self-evaluations of happiness, satisfaction, and meaning [26]. 

Building on this perspective, the OECD *Guidelines on Measuring Subjective Well‑Being* identify three key components of subjective well‑being: life evaluation, affect, and eudaimonia [26]. Life evaluation refers to a reflective assessment of one’s life as a whole or specific domains within it, such as work or relationships [26]. Affect captures emotional states, including both positive and negative feelings, often tied to a particular moment in time; this dimension is commonly associated with hedonic well-being, focused on pleasure and avoidance of pain [26]. Eudaimonic well-being, by contrast, emphasizes a sense of meaning, purpose, and psychological flourishing, often linked to concepts like belonging and personal growth. Together, these dimensions represent complementary aspects of subjective individual well-being: evaluation, emotion, and purpose [26]. 

Individual well-being is also shaped by contextual conditions that support or constrain people’s ability to live well. Terms such as *flourishing* or *thriving* are sometimes used to describe this broader perspective, emphasizing the role of environments that enable individuals to grow and function optimally [23]. An individual’s well-being is inherently connected to the well-being of their community, as people both contribute to and depend on collective conditions that sustain quality of life [27]. In this way, more expansive definitions of well-being link personal fulfillment with the social and physical environments that support it [27]. 

Conceptualizing community well-being goes beyond simply aggregating the well-being of individuals, though the two are closely connected. Frameworks in the community well-being literature describe a healthy or thriving community as dependent on the well-being of its members, as well as by strong relationships, effective leadership, equitable practices, and a shared sense of purpose [28]. Many models focus on how community characteristics influence individual experiences, yet this perspective can overlook broader structural factors, including social and economic inequality, local context, and systemic challenges. An alternative approach views community well-being as a collective process of “being well together,” in which relationships, institutions, and community dynamics collectively shape shared quality of life. In newer work, this approach is separated out as *collective well-being* [28]. 

For the purpose of this article, we adopt a definition of *community well-being* developed by the Robert Wood Johnson Foundation: “the combination of social, economic, environmental, cultural, and political conditions identified by individuals, families, and their communities as essential for them to flourish and fulfill their potential.” [29].

### Mindsets and well-being perceptions

Understanding *mindsets* related to individual and community well-being is a critical element of advancing the measurement and promotion of community well-being [19–21]. These mindsets—people’s underlying beliefs and assumptions about what drives well-being, who is responsible for it, and how it can be improved—extend the concept of *health mindsets* that shape expectations, behaviors, and support for policies promoting health. Subjective well-being assessments, and specifically “well-being perceptions” as part of that, can be viewed as one element of these mindsets, reflecting how individuals interpret their life circumstances and the broader conditions of their communities [30]. These perspectives provide essential context for designing initiatives that resonate with community priorities and for interpreting traditional indicators—such as economic or health metrics—within the lived experiences of individuals [28]. Incorporating these subjective perspectives can improve the validity and relevance of well-being measures and ensure that efforts to enhance well-being are informed by what people think matters most [19]. 

Studies show that people frequently distinguish between their personal well-being and their perceptions of community well-being [28]. For example, someone may report high life satisfaction while perceiving their community as struggling due to issues like limited resources, social inequality, or weakened social cohesion. This distinction aligns with the concept of egocentric versus sociocentric evaluations, where individuals assess their personal circumstances differently from broader societal or community conditions [31]. Such differences are often influenced by geographic and social contexts, with urban residents tending to perceive their communities as resource-rich but less cohesive, while rural residents report stronger social ties but more limited access to resources [32]. 

Social comparison theory also sheds light on how individuals evaluate their well-being relative to others in their community: Festinger (1954) posited that people’s perceptions of their personal circumstances are shaped by comparisons with those around them, a process that can extend to how groups collectively assess community conditions [33]. In the context of community well-being, these evaluations may draw not only on individual experiences but also on shared narratives about local opportunities, fairness, and quality of life for others in the community [28]. For example, intersubjective assessments, as described by Lee and Kim (2016), ask people to evaluate whether their members of their community are satisfied overall, rather than focusing solely on their own satisfaction [34]. Through these shared assessments, patterns of comparison and communication among residents can give rise to common perceptions of how well the community is functioning. These collective evaluations often center on widely recognized dimensions of social life, including trust among neighbors, perceptions of safety, and a sense of belonging or social cohesion, that reflect the quality of relationships and institutions within a place and have been consistently linked to higher reported community well-being [24, 35, 36]. 

### Current state of well-being measurement

The literature includes several examples of instruments and scales to measure individual well-being [23, 26, 37, 38], validated and intended for use in various settings. Most existing measures focus on subjective individual well-being, with the Cantril Ladder being a commonly used tool for assessing overall life evaluation in population surveys [39]. The Cantril Ladder is recommended by the OECD and is used in major nationally representative studies such as the Global Flourishing Study (GFS) and the Gallup Global Well-being Index and the Gallup World Poll, which informs the World Happiness Report. The Cantril Ladder is scored on a scale of 0 to 10, and according to Wave 1 data (2022–2024) of the GFS, the average Cantril Ladder score for Americans is 7.46 (SE = 0.008) [27], while the Gallup World Poll reports a U.S. average of 6.96, with the U.S. ranking 24th globally over a three-year period [40]. 

However, measurement efforts have shown that Americans tend to be more optimistic about their personal futures than about the future of their communities or the nation: while 84% of Americans feel hopeful about their own future, only 76% feel hopeful about the future of their communities, and just 52% are hopeful about the nation’s trajectory over the next few years [41]. While there are many ways to interpret the difference between these values, these gaps highlight the importance of measuring both personal and community dimensions of well-being rather than relying sole on individual measures in order to gain a nuanced understanding of community well-being.

As noted earlier, community well-being reflects the social, economic, environmental, cultural, and political factors that people see as essential for thriving. While community well-being measurement is still evolving, a range of approaches have been developed in research, government, and nonprofit settings [42–52]. Most frameworks recommend combining subjective and objective indicators at both individual and community levels, including satisfaction with life, perceptions of community characteristics, and objective measures such as education, healthcare access, and public safety [42, 43]. Newer frameworks, such as the Well-Being in the Nation Measurement Framework and the Community Well-being Atlas, recommend integrating qualitative, quantitative, and geographic data, as well as contextual indicators defined by community members, to ensure measures reflect local priorities and differences and integrate elements of equity [44–46]. Intersubjective measures—assessing how residents perceive others’ well-being—along with collective narratives and deliberative forums, further enrich the ability of these approaches to speak to different aspects of community well-being [43, 53]. 

A growing number of communities are adopting some of these recommendations to better understand and improve the conditions that support community well-being. In the United States, cities such as Santa Monica, California; Tacoma, Washington; Louisville, Kentucky; Nashville, Tennessee; Green Bay, Wisconsin; and Atlanta, Georgia have implemented well-being initiatives tailored to their unique local contexts [47–52]. These efforts have included measures of physical and mental health, education, economic opportunity, and occasionally social connections. For example, Santa Monica’s Wellbeing Index emphasizes community-level conditions and connections, such as access to resources and civic participation, rather than simply aggregating individual well-being scores [48]. Nashville and Atlanta have focused on using well-being data to guide policy decisions and investments in areas like public health and economic equity [50, 51]. 

Globally, countries including Canada, New Zealand, Scotland, Germany, Wales, Italy, and Iceland have implemented national well-being measurement systems [54–60]. New Zealand in particular has emerged as a leader in national-level well-being measurement, incorporating indicators such as mental health, environmental sustainability, and governance into its policy framework [54]. Yet most of these local and national initiatives continue to focus primarily on individual-level outcomes or broad population indicators. Only a few explicitly assess the social and structural factors that make communities health-promoting or thriving, such as civic participation, inclusion, and local capacity to meet residents’ needs [48, 54]. As a result, systematic measurement of people’s perceptions of community-level well-being remains limited, underscoring a need to capture these collective conditions more directly. And while these efforts mark a significant shift toward more comprehensive well-being measurement, many remain limited by infrequent data collection and short-lived initiatives. Sustained, consistent measurement that addresses both conditions and connections within communities can inform long-term strategies to address systemic challenges and foster thriving communities [46]. 

### Contributions of this paper

This paper uses data from the 2023 National Survey of Health Attitudes (NSHA) to explore elements related to individual and community well-being, addressing key gaps in the literature on how people perceive the well-being of their communities. While measures of individual well-being are well-established and easily collected, assessing community well-being is more complex. Existing approaches in communities, as described previously, often rely on aggregated individual evaluations or overly broad questions, and tend to miss specific conditions and social connections that enable communities to thrive. They also provide examples of objective indicators of community well-being but lack a recognition of the importance of subjective indicators (e.g., perceptions as a representation of mindsets). This study advances the field by offering insights into mindsets related to community well-being on a national scale and offering recommendations to help local leaders understand public sentiment and target improvements more effectively. This study provides new insights into the factors that shape perceptions of community well-being and their relationship to individual well-being. This work also deepens understanding of the interplay between personal circumstances and factors at the community level, supporting more effective measurement and intervention strategies.

For leaders in local communities interested in advancing community well-being, this paper highlights the importance of selecting sentinel measures that are actionable and forward-looking—that is, indicators that not only reflect current conditions but also signal emerging trends and suggest future trajectories within communities (e.g., support for youth) [7]. Effective measurement depends on using comprehensive and connected data that capture the multiple dimensions of well-being, including access to resources, social connections, and environmental factors. By focusing on multidimensional measures that reflect aspects of both individual and community well-being, local leaders can design initiatives that resonate with residents and support coordinated efforts to improve quality of life.

### Study aims

This study seeks to deepen our understanding of how Americans conceptualize and evaluate community well-being to contribute to a wider body of work on health mindsets [20–22, 61]. By examining perceptions of community well-being alongside subjective individual well-being, demographic factors, and specific elements of community conditions and connections, the analysis provides insight into the evaluative beliefs and assumptions that shape broader mindsets about what constitutes well-being for individuals and communities. Key research questions include:

- How do perceptions of community well-being relate to individuals’ perceptions of their own well-being?
- How do perceptions of individual and community well-being differ by demographic and economic characteristics?
- What specific elements of community well-being are most related to perceptions of overall community well-being?

## Methods

### Survey

This study relies on data from the 2023 NSHA, funded by the Robert Wood Johnson Foundation and previously fielded in 2015 and 2018. The NSHA is a nationally representative survey designed to capture public perspectives and mindsets on various topics related to health and well-being. The 2023 survey is the first wave of the survey to include questions on both individual and community well-being, offering new and valuable insights into how Americans perceive these dimensions. Data collection took place between November 27 and December 19, 2023 via two existing survey panels: the RAND American Life Panel (ALP) and the Ipsos KnowledgePanel, yielding a final sample of 5,620 respondents, with 1,570 participants from the ALP and 4,050 from the KnowledgePanel. ALP and KnowledgePanel panel participants complete surveys online, and both panels provide computers and Internet connections for respondents who do not already have them. Survey respondents were compensated for their time based on the length of the survey [61]. Samples were weighted using a raking algorithm prior to analyses to be representative of the U.S. population in terms of gender, race/ethnicity, education, age, income, and household size, using data from the 2022 Current Population Survey to characterize the U.S. population [22]. Detailed information on the origins of the survey can be found in Carman, et al., 2016 [61] and additional methodological information on the two panels, how data were collected, the NSHA sample, and more detail on approaches to weighting are available in Chandra, et al., 2024 [22]. 

### Measures of well-being

Analyses presented in this paper rely on 15 survey questions from the 2023 NSHA which focus on individual and community well-being. *Individual well-being* was assessed with the Cantril Ladder [62], a widely-used measure of subjective individual well-being which presents a picture of a ladder with the prompt: “Assume the ladder is a way of picturing your life. The top of the ladder represents the best possible life for you and the bottom of the ladder represents the worst life for you. On which step of this ladder would you say you personally feel you stand at this time?” and asks participants to rate their lives on a scale of 0 to 10, where 0 is the worst possible life for you and 10 is the best possible life for you.

Perceptions of *community well-being* were assessed in two ways: (1) an overall rating of community well-being and (2) via a series of 13 questions about specific elements that contribute to community well-being. Respondents were asked to rate the overall well-being of the community in which they live on a five-point scale from “Poor” to “Excellent” (henceforth the “overall community well-being”). The survey item referenced the “community in which you live” without further elaboration or definitional prompts. This approach permitted respondents to interpret the construct of “community well-being” according to their own experiential and contextual understanding. This approach is consistent with established practice in exploratory place-based and community well-being research [26, 63, 64]. This approach also aligns with evidence that definitions of community vary widely according to geographic, social, and identity-based boundaries, and that respondents’ lived experiences inform their perceptions of “community well-being” [65, 66]. 

Specific elements that contribute to community well-being were assessed through measures in two domains of community conditions and community connections, capturing both structural resources and social dynamics. Community conditions items focused on *perceptions* of the degree to which everyone in the community has access to health care, healthy food, safe drinking water, outdoor spaces, affordable housing, and multi-modal transportation infrastructure. Community connections items examined *perceptions* of the connections between community members and between residents and decisionmakers through questions about diversity celebration, community-wide events, trust, mutual support during times of need, familiarity among members, collaboration for health, and participation in decision-making. Respondents rated their agreement with statements about each of these 13 aspects of their community on a four-point scale from “Not at all” to “Completely.” (Text of all survey items used to measure individual and community well-being can be found in the Supplementary Information.) Together, these measures provide a holistic view of perceptions of the physical and social factors contributing to community well-being.

### Analysis

We began our analysis by calculating descriptive statistics (e.g., frequencies, means) by different respondent characteristics for our measures of interest to detect initial patterns and inform subsequent analyses. For individual well-being, in addition to calculating means, scores on the Cantril Ladder were grouped in a way analogous to the Gallup Life Evaluation Index, with respondents classified as “thriving” (7 and above), “struggling” (5 or 6), or “suffering” (4 and below) based on how they rate their current lives.^1^ To explore relationships between individual well-being, elements of community well-being, and overall community well-being, we conducted two sets of analyses: correlations and logistic regression models. All analyses were conducted in Stata 17.0.

#### Correlational analysis

Using Spearman correlation, we examined the association between individual well-being (i.e., Cantril Ladder) and overall community well-being. We also examined correlations between individual well-being and specific community well-being elements, all dichotomized as “Not at all/Somewhat” vs. “Mostly/Completely.” Additionally, we assessed correlations between overall community well-being and these same community elements. These analyses offered insight into how perceptions of individual well-being relate to distinct aspects of community well-being, including community conditions that provide access to resources (e.g., health care, housing, transportation) and social factors that relate to community connections (e.g., trust, mutual support, and collaboration).

#### Logistic regression analysis

We conducted logistic regression analysis to investigate the predictors of overall community well-being using dichotomized elements of community well-being and sociodemographic factors as independent variables and also controlling for sociodemographic variables. The overall community well-being measure was dichotomized as “Fair/Poor” vs. “Good/Very Good/Excellent”. Sociodemographic and other control variables included sex, race, income, age, education, urbanicity, and survey sample (ALP or KnowledgePanel) allowing for the assessment of perceptions of community-level factors independent of these characteristics. The reference groups for control variables were non-Hispanic white, male, annual household income less than $10,000, age under 25, less than high school education, and rural. Each model estimated the odds of rating overall community well-being positively based on sociodemographic factors and the presence of specific community conditions (e.g., access to health care, safe drinking water, affordable housing) and connections (e.g., trust, mutual support, diversity celebration). The presence of each condition is assessed here as “mostly” or “completely” true about respondents’ community vs. “not at all” or “somewhat”. We also ran a goodness of fit test using an F-adjusted mean residual test on our dichotomized response model of survey data. These analyses aimed to uncover the elements of community well-being most related to perceptions of overall community well-being while accounting for individual demographic factors.

#### Additional analyses

As a sensitivity check, we also estimated an ordered regression model using the full ordinal scale for overall community well-being as the dependent variable (see Appendix for results of the ordered logistic regression). Patterns of association in these models were generally consistent with those observed in the dichotomized analyses, indicating robustness of the findings to model specification. We elected to present results from dichotomized logistic regression models because the binary outcome clarifies effect estimates as the odds of reporting positive (“Good,” “Very Good,” or “Excellent”) community well-being versus less-positive (“Fair” or “Poor”). Dichotomizing the scale in this exploratory analysis enabled a clear comparison between more positive and less positive perceptions and improved result interpretability. This approach is consistent with prior research using dichotomization in early-stage or policy-oriented analyses to simplify communication and highlight meaningful distinctions in perceptions [26, 67]. For transparency regarding the incremental contributions of variable blocks (sociodemographic variables, community conditions, community connections), we conducted a set of stepwise regression models (results in the Appendix). Finally, we conducted an exploratory factor analysis of the 13 community well-being element items to assess whether they reflected underlying latent dimensions that contribute to perceptions of overall community well-being. Although fit indices supported a four-factor solution (RMSEA = 0.055, CFI = 0.992, TLI = 0.980, SRMR = 0.017), the pattern of item loadings lacked a clear conceptual structure, and thus no composite factor scores were used in subsequent analyses.

## Results

Table 1 of the unweighted survey sample shows that our sample was roughly evenly split by gender and approximately half Non-Hispanic white. Annual household income was over $50,000 per year for over two-thirds of our sample. Almost two-thirds of the sample was over 45 years old, and the majority had attended at least some college. (Final survey data were weighted to be representative of the U.S. population prior to conducting other analyses.) Finally, our sample lived primarily in urban areas or communities with populations over 50,000 people. (Urbanicity will henceforth be described as “urban” and “rural” communities.)

**Table 1:** Survey sample characteristics

Characteristic	Unweighted %
Total sample	*N* = 5,620
Gender	
Male	48.4
Female	51.6
Race or ethnicity	
Non-Hispanic white	50.4
Non-Hispanic Black	20.0
Hispanic	21.6
Non-Hispanic, Asian/Pacific Islander	5.7
Non-Hispanic, other	2.3
Annual household income, in U.S. dollars	
Less than 10,000	3.5
10,000 to 24,999	7.9
25,000 to 49,999	17.3
50,000 to 74,999	18.6
75,000 to 99,999	13.0
100,000 or more	39.7
Age, in years	
18–24	5.4
25–44	26.8
45–64	35.7
65+	32.0
Education	
Less than high school	6.9
High school	21.9
Some college	29.0
College graduate	22.9
Postgraduate	19.3
Urbanicity	
Rural: Rural/small town	14.2
Urban: City, population 50k+	85.8

**Fig. 1:**
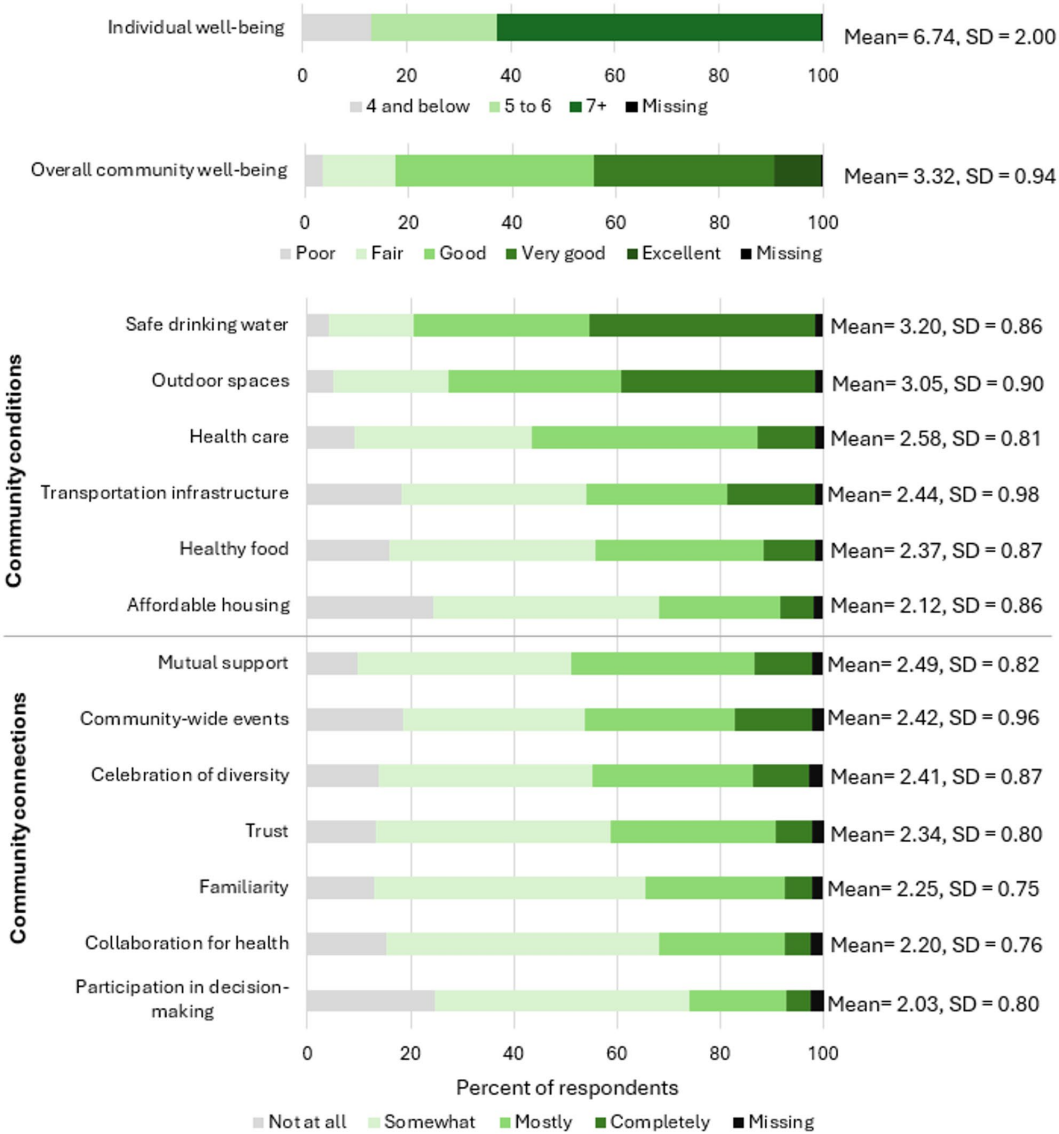
Descriptive results of all well-being measures. Individual well-being measured with Cantril Ladder: “Assume the ladder is a way of picturing your life. On which step of this ladder would you say you personally feel you stand at this time? 0 is the worst possible life for you, 10 is the best possible life for you.” Individual well-being responses grouped per methodology developed by Gallup to summarize results of the Cantril Ladder. [68] Overall community well-being measured with the survey question: “How would you rate the well-being of the community in which you live?” and answers were coded on a scale of 1 to 5. Elements of community well-being were coded on a scale of 1 to 4

Descriptive analyses revealed differences in perceptions of individual and community well-being, as well as specific elements of community conditions and connections (Fig. 1). Most respondents rated their individual well-being relatively high, with 68% scoring seven or higher on the Cantril Ladder (mean = 7.01, SE = 0.025). Overall community well-being also received generally positive ratings, with around 44% rating it as “Excellent” or “Very Good” and around 17% as “Fair” or “Poor.”

Among elements of community well-being, access to safe drinking water and outdoor spaces were rated the highest, with means of 3.22 and 3.07 out of five, respectively, while affordable housing and participation in decision-making received the lowest ratings, with means of 2.15 and 2.04, respectively. These findings suggest that, although individuals generally rate both their own well-being and the overall well-being of their community positively, perceptions of whether key elements that support community well-being are present in their community are more variable.

### Exploration of individual well-being

Descriptive analyses of individual well-being revealed notable variation across income, age, education, race/ethnicity, and urbanicity (Appendix Table A1). Higher well-being scores were consistently and significantly associated with greater household income, older age, and higher educational attainment. For example, respondents earning $100k or more, aged 65+, or holding postgraduate degrees had the highest mean well-being scores, and generally, individual well-being increased as income, age, or educational attainment increased. Differences by race/ethnicity were statistically significant and showed Non-Hispanic white respondents reporting the highest mean well-being, while rural residents exhibited statistically significantly higher levels of individual well-being than urban residents.

### Exploration of community well-being

Our primary focus is regression results (Table 2) but we report correlations in Appendix Table A2 without significance testing, for foundational information. Individual well-being was weakly positively associated with perceptions of overall community well-being (correlation coefficient = 0.291). Elements of community well-being showed weak positive associations with individual well-being (range of correlation coefficients = 0.053–0.196) and weak to moderate positive associations with overall community well-being (range of correlation coefficients = 0.119–0.397).

**Table 2:** Ratings of overall community well-being as a function of demographics and specific elements of community well-being

Factors	Odds ratio (95% CI)
Race/ethnicity (Reference group: NH white)	
NH Black	0.814* (0.652–1.016)
Hispanic	0.885 (0.709–1.106))
NH Asian/PI	1.604** (1.014–2.537)
NH other	0.600** (0.367–0.982)
Gender (Reference group: male)	
Female	0.976 (0.827–1.151)
HH income (Reference group: <$10k)	
$10k-25k	1.139 (0.755–1.719)
$25k-50k	1.401* (0.955–2.057)
$50k-75k	1.835*** (1.238–2.719)
$75k-100k	1.806*** (1.189–2.744)
$100k+	2.960*** (1.985–4.413)
Education (Reference group: < HS)	
HS graduate	1.093 (0.801–1.491)
Some college/associate’s degree	1.153 (0.840–1.582)
College graduate	1.200 (0.842–1.711)
Master’s degree or above	0.944 (0.652–1.366)
Urbanicity (Reference group: rural)	
Urban	1.431*** (1.131–1.809)
Age (Reference group: < 25)	
25–44	0.553*** (0.370–0.828)
45–64	0.535*** (0.358–0.800)
65+	0.903 (0.591–1.378)
Survey sample (Reference group: KnowledgePanel)	
ALP	0.833* (0.678–1.024)
Elements of community well-being	
*Conditions*:	
Health care	1.394*** (1.144–1.698)
Healthy food	1.671*** (1.332–2.096)
Safe drinking water	1.235** (1.016–1.503)
Outdoor spaces	2.498*** (2.060–3.030)
Affordable housing	1.123 (0.887–1.421)
Transportation infrastructure	0.766*** (0.635–0.926)
*Connections*:	
Celebration of diversity	1.107 (0.892–1.375)
Community-wide events	1.012 (0.813–1.261)
Trust	2.081*** (1.597–2.711)
Mutual support	1.735*** (1.370–2.197)
Familiarity	0.841 (0.660–1.072)
Collaboration for health	1.316** (1.004–1.727)
Participation in decision-making	1.071 (0.812–1.411)

*NH* Non-Hispanic, *HS* High school

*** *p* < 0.01, ** *p* < 0.05, * *p* < 0.1. For analysis of elements of well-being, we controlled for race/ethnicity, gender, income, education, urbanicity, survey sample, and age

Logistic regression analyses revealed significant associations between overall community well-being ratings and some sociodemographic factors, as well as specific elements of community well-being (Table 2). An F‑adjusted mean residual test indicated that the model fit the data adequately, F(9, 5,344) = 1.50, *p* = 0.140.

Among sociodemographics, Non-Hispanic Asian respondents were more likely to report more positive community well-being ratings (OR = 1.604, 95% CI: 1.014–2.537) and those in the “Non-Hispanic Other” racial/ethnic group were less likely to report more positive community well-being (OR = 0.600, 95% CI: 0.367–0.982). Higher household income was strongly associated with more positive community well-being ratings, with respondents earning $100k or more exhibiting over three times the odds (OR = 2.960, 95% CI: 1.985–4.413) of rating their community well-being favorably compared to those earning less than $10k. Urban residents were also more likely to rate their community well-being positively (OR = 1.431, 95% CI: 1.131–1.809), while respondents aged 25–44 and 45–64 had significantly lower odds of rating their community well-being as “very good” or “excellent” compared to those under 25.

Specific elements of community well-being demonstrated strong predictive power. Among community conditions, outdoor spaces for physical activity had the strongest positive association with favorable community well-being ratings (OR = 2.498, 95% CI: 2.060–3.030), followed by access to healthy food (OR = 1.671, 95% CI: 1.332–2.096), access to health care (OR = 1.394, 95% CI: 1.144–1.698), and access to safe drinking water (OR = 1.235, 95% CI: 1.016–1.503). In contrast, transportation infrastructure was negatively associated with positive community well-being ratings (OR = 0.766, 95% CI: 0.635–0.926).

For community connections, trust among community members emerged as a key predictor of favorable ratings (OR = 2.081, 95% CI: 1.597–2.711), along with mutual support in times of need (OR = 1.735, 95% CI: 1.370–2.197). Collaboration for health also showed a weaker positive association (OR = 1.316, 95% CI: 1.004–1.727), whereas other connection elements, such as celebration of diversity, familiarity with other community members, and participation in decision-making, did not significantly predict overall community well-being.

## Discussion

### Interpretation of results

Descriptive analyses revealed that individual well-being was rated relatively high overall, with 68% of respondents with “thriving” ratings (Cantril Ladder scores over 7). However, there were clear sociodemographic differences, with individual well-being increasing across income, age, and education levels. For instance, respondents earning $100k or more, aged 65+, or holding postgraduate degrees had the highest mean well-being scores, while those in lower income brackets, younger age groups, or with less formal education reported lower scores. These findings are consistent with patterns observed in prior research among American adults. Higher income and greater educational attainment are often associated with increased access to resources, stability, and opportunities that can support health and life satisfaction [69]. Likewise, older adults may report higher well-being because they have greater emotional regulation and life experience, as well as different expectations and priorities that contribute to a more favorable evaluation of their lives [11, 12, 70]. Overall, individual well-being scores described in this paper, measured using NSHA data in 2023 (mean = 7.01) were slightly higher than the U.S. average calculated from the 2024 Gallup World Poll (mean = 6.96), but lower than the average reported by the Global Flourishing Study for 2022–2024 (mean = 7.46).

Individual well-being was not significantly associated with perceptions of overall community well-being, suggesting that respondents may distinguish between their personal circumstances and broader community-level conditions. This is consistent with prior work in political science examining differences in egocentric and sociocentric assessments, which has found that individuals may base evaluations of their community on shared resources, public services, and collective conditions rather than on their own personal circumstances [31]. Ratings of overall community well-being varied significantly by sociodemographics, particularly household income and urbanicity. Higher income was strongly associated with positive community well-being ratings, with individuals earning $100k or more exhibiting over three times the odds of favorable ratings compared to those earning less than $10k. Prior research has shown that reported built and social environmental amenities are positively associated with neighborhood income [71]. Urban residents were also significantly more likely to rate their community well-being positively, which may reflect greater access to resources and opportunities in urban areas; [72, 73] however, there is unexplored nuance to this finding, as rural communities are diverse and possess unique strengths that may not be captured in responses to broad-strokes community well-being measures [74]. Respondents in midlife (ages 25–64) were significantly less likely to report positive community well-being compared with younger and older adults. There is limited existing literature on perceptions of community well-being over the life course. Midlife adults often face competing work, caregiving, and financial demands that reduce time for community participation and may weaken social connectedness, factors that may influence perceptions of local quality of life and collective efficacy [75–77]. They may also experience a mismatch between expectations for community responsiveness and the realities of local resources or governance [76], potentially leading to more critical evaluations of community well-being.

Logistic regressions uncovered which elements of community well-being were significantly associated with respondents’ overall perceptions of their communities. In other words, they provide insight into which elements of their communities might make respondents feel like those communities have the conditions necessary for thriving. Elements within both conditions and connections categories emerged as important predictors of overall community well-being ratings. Within community conditions, outdoor spaces had the strongest positive association, suggesting that safe and accessible places for physical activity are a critical component of community well-being. Access to healthy food also showed significant positive associations. These findings align with the broader literature on the social drivers of health, which highlights the role of physical environments and access to resources in shaping health and well-being outcomes [78]. States and local governments are also increasingly incentivizing community designs that promote physical activity and address food deserts as part of broader health equity efforts [79, 80]. By identifying access to healthy foods and opportunities for physical activity as important components of community well-being, these results suggest that these types of investments align with respondents’ priorities for community well-being and can directly inform incentive program planning, helping states and local governments focus resources on changes most likely to be supported and utilized by the community.

Access to healthcare was also a predictor of positive perceptions of community well-being, indicating that it continues to play a central role in the health and well-being narrative and reflecting its enduring importance in public perceptions of health determinants. Over the past almost two decades, survey respondents have ranked factors such as access to affordable healthcare, health insurance coverage, and personal health practices as the most influential perceived drivers of well-being (even above the social drivers of health), highlighting the dominance of healthcare services and personal choice in shaping public attitudes about health [81]. As noted in a Robert Wood Johnson Foundation report on community health narratives [82], beliefs about the relative importance of healthcare services versus social drivers of health also remain a critical point of discussion at the community level, underscoring the need to balance these perspectives when addressing health equity and well-being.

Connections within communities also played a significant role in shaping perceptions of community well-being. Trust among community members was the strongest predictor of favorable ratings, followed by mutual support in times of need and collaboration for health. These findings are supported by prior research showing that communities with strong relationships and mutual trust are better able to adapt to challenges and support the well-being of their members (i.e., they have higher community resilience) [83]. Supportive community relationships can amplify the benefits of physical environments on well-being, and also provide access to critical resources in times of need, such as information, aid, and emotional support [84]. These findings suggest that fostering strong social networks, cohesion, and trust within communities may be as important as addressing physical infrastructure when aiming to improve community well-being [44, 85]. Furthermore, there is a role for physical infrastructure to support social connection, as highlighted in the place-making literature [86]. These measures also highlight aspects of connection within communities that are important elements within the emerging concept of collective well-being.

Although many community well-being elements demonstrated significant positive associations with overall community well-being, some displayed weaker or counterintuitive patterns that merit closer examination. For example, transportation infrastructure was negatively associated with favorable community well-being ratings—a surprising result given its potential to enhance mobility and access to resources. This may be explained by residents’ experiences with public transportation, as factors such as perceived safety when walking at night and long commute times can negatively affect satisfaction with transit systems [87, 88]. Additionally, access to safe drinking water, while positively associated with overall community well-being, was also rated highly across respondents, suggesting that it may be less salient as a differentiating factor in perceptions of community well-being. These findings highlight the need for more nuanced examinations of how specific community characteristics or personal attitudes interact with broader perceptions of well-being.

Despite the documented importance of several elements of community well-being, some findings from this study revealed gaps between evidence and public perceptions. Presence of affordable housing, for example, was an element of community well-being with one of the lowest ratings, and connections between housing and community well-being were not readily recognized by respondents, despite substantial evidence linking stable, affordable housing to improved health and well-being outcomes [89, 90]. This is consistent with prior work that found that survey participants did not organically connect affordable housing with improved health [91]. While trust among community members and mutual support was linked to community well-being appraisals, broader elements of social cohesion and engagement, such as celebration of diversity and inclusion, community events, familiarity among neighbors, and participation in decision-making, did not emerge as significant predictors of community well-being. Prior studies from the participatory democracy literature have highlighted the importance of shared spaces, community amenities, and community-informed decision-making as critical to cultivating community belonging, yet our findings show these concepts may be less prominent as part of mindsets about community well-being [44]. Bridging these gaps will require targeted efforts to raise awareness of the role of investments and initiatives that promote housing stability, social cohesion, and participatory governance in supporting well-being.

### Application for leaders in local communities

Findings from this work may be of interest to leaders in local communities grappling with declining well-being and looking for better ways to keep a pulse on public sentiment and whether policies and investments are appreciably improving peoples’ lives. As communities increasingly prioritize well-being in policy documents and local strategies, the need for robust and multidimensional measures of community well-being becomes critical. Our study contributes to the literature by examining not only multiple dimensions—such as social connections, access to resources, and environmental conditions—but also the relationship between individual and community well-being. By integrating both perspectives, we provide a more comprehensive understanding of how well-being is experienced and perceived, and why it is important to measure well-being at multiple levels, and examine connections between those levels, rather than relying solely on aggregated individual data or single subjective indicators.

Multidimensional measures can help leaders identify specific drivers of well-being and tailor interventions to address local priorities. For example, our findings indicate that outdoor spaces, trust, and mutual support were positively associated with community well-being. While these relationships highlight areas that may be relevant for community initiatives, the cross-sectional analyses do not allow for causal inference. Tools for community leaders like the ALIGN for Health and Well-being Toolkit provide transformative examples of how communities are linking well-being measures to actionable strategies, helping leaders align narratives about community priorities with targeted investments [7]. Forward-looking measures, such as those focused on community connections or investments in health-promoting amenities, can also provide leaders with a way to monitor progress toward future thriving, ensuring that investments remain relevant and effective.

Additionally, integrating data systems across sectors is essential to fully capture the interconnected factors that shape well-being. By breaking down silos in data collection and analysis—moving beyond program-specific or topic-specific metrics—communities can develop a more comprehensive understanding of how different elements, such as housing, transportation, and health, interact to influence well-being. This approach allows for more coordinated strategies that address multiple priorities simultaneously, such as improving access to affordable housing while also enhancing health equity and social cohesion.

The next frontier for communities is grappling with the concept of *collective* well-being, which emphasizes the shared sense of connection and mutual support among individuals, institutions, and the environment within a community, enabling people to work together to address challenges and build a better future. Early work on collective well-being has shown that collective well-being is shaped by shared experiences, such as those during the COVID-19 pandemic, where perceived well-being influenced both individual and community outcomes [92]. By incorporating this perspective, leaders can design policies that foster stronger connections among individuals, institutions, and the environment.

Ultimately, integrating multidimensional measures into decision-making processes enables local leaders to understand what truly makes a difference in people’s lives. This approach moves beyond traditional economic metrics that are often used to characterize communities, offering deeper insights into the factors that contribute to thriving communities. By focusing on these measures, leaders can ensure that policies and investments resonate with residents’ lived experiences, fostering communities where people thrive collectively.

### Strengths and limitations

This study benefits from its nationally representative sample, allowing for robust insights into individual and community well-being across diverse demographic groups. A particular strength is the ability to differentiate community well-being—defined as the combination of social, economic, environmental, cultural, and political conditions essential for individuals and communities to flourish—from an aggregation of individual well-being. Additionally, the study’s design enables the analysis of specific community elements, providing valuable insights into the importance of various factors, such as outdoor spaces and trust, in shaping perceptions of community well-being.

However, several limitations should be noted. The study is observational and cross-sectional and cannot establish causal relationships between community elements and community well-being, and the interrelated nature of community elements makes it challenging to fully disentangle their effects. The study did not collect detailed information about respondents’ specific communities—such as geographic location, local conditions, or contextual factors like public safety, employment opportunities, and economic prosperity, beyond a general classification of community size (i.e., “urban” for populations greater than 50,000 and “rural” for smaller communities). The survey also did not include measures of some individual characteristics, such as political affiliation, that may shape perceptions of community well-being. In addition, the survey did not provide a formal definition of “community,” allowing respondents to interpret the term in their own ways, complicating interpretation of the findings. The study also introduces questions about equitable access, as survey questions asking whether everyone in the community has “access to” certain amenities inherently touch on equity issues but do not delve deeper into how access is distributed across different groups.

While this study was primarily focused on community well-being, the reliance on the Cantril Ladder to measure individual well-being, while widely used, has been critiqued for its limitations in capturing nuanced aspects of well-being, including focusing too much on power and wealth rather than broader aspects of well-being and missing broader cultural contexts including how life appraisal is situated against group membership and the treatment of that group in society [39]. Moreover, the study does not measure hope for the future—a critical dimension of well-being that reflects optimism and collective aspirations, particularly in the face of systemic challenges. While individual well-being was not the focus of this analysis, measures like the WHO-5 Well-being Index or the Ryff Scale of Psychological Well-Being could have captured other important dimensions [93]. (Other limitations related to the survey sample, design, and weighting for the NSHA are described in Chandra, et al., 2024.) Data were collected in 2023, and the intervening years have been marked by significant national and global events, which may have shaped perceptions and contributed to discrepancies with other indices, such as results from Gallup’s World Poll. The choice to use logistic regression to analyze relationships between elements of community well-being was chosen to aid interpretability of the results, though the dichotomization required to use this approach sacrifices some of the richness offered by the full-scale results. Finally, the study does not directly address the emerging concept of collective well-being, which emphasizes the interconnectedness of individual and community well-being and the collective capacity to address systemic challenges and build shared futures. Incorporating collective well-being and hope for the future into future research could provide deeper insights into the dynamics of well-being.

## Conclusions

This study provides key insights into individual and community well-being in the United States, highlighting the conditions and connections that are related to more positive perceptions of community well-being. Elements commonly emphasized in public health and community resilience research were salient to perceptions of community well-being, including access to amenities that support physical activity, availability of healthy food, and the presence of trust and mutual support. These findings suggest a critical role for both physical and social infrastructure in fostering community well-being. For local leaders, these findings underscore the need for multidimensional measures that capture perceptions of specific elements of community well-being in addition to measures of individual subjective well-being. Integrating data systems across sectors and adopting forward-looking, sentinel measures can help leaders anticipate needs and evaluate the impact of policies over time.

## Supplementary Information

Supplementary Material 1.

## Data Availability

The dataset supporting the conclusions of this article is available in the Inter-university Consortium for Political and Social Research (ICPSR) repository, [https://www.icpsr.umich.edu/web/HMCA/studies/39205/versions/V1](https://www.icpsr.umich.edu/web/HMCA/studies/39205/versions/V1) [94].

## Abbreviations

ALP: American Life Panel
CI: Confidence interval
HS: High school
GFS: Global Flourishing Study
NH: Non-Hispanic
NSHA: National Survey of Health Attitudes
OECD: Organisation for Economic Co-operation and Development
OR: Odds ratio
SE: Standard error

## Footnotes

1 For Gallup’s Life Evaluation Index, Gallup classifies Americans as “thriving,” “struggling” or “suffering” according to how they rate their current *and future* lives on the Cantril Ladder. Our study differed in that we only asked respondents to evaluation their current individual well-being using the Cantril Ladder, but numeric groupings were consistent with the Gallup approach.
