# Supplementary material for: Examining the American mindset on community well-being: insights from a national survey
Source: BMC Public Health. 2026 Feb 11;26:902. doi: 10.1186/s12889-026-26507-0 (PMC12998088; doi:10.1186/s12889-026-26507-0)
Supplement: Supplementary file 1 — Supplementary Material 1. [file 12889_2026_26507_MOESM1_ESM.docx]

**Appendix to “Examining the American Mindset on Community Well-Being: Insights from a National Survey”**

All NSHA individual and community well-being survey items used in analysis are as follows:

Individual well-being:

“Assume the ladder is a way of picturing your life. On which step of this ladder would you say you personally feel you stand at this time? 0 is the worst possible life for you, 10 is the best possible life for you.”

Community well-being:

- Overall community well-being:

“How would you rate the well-being of the community in which you live?” (5-point scale, Poor to Excellent)

- Community conditions (all use a 4-point scale for rating agreement with each question, Not at all to Completely):
  - “Everyone in my community has access to needed health care and services.”
  - “Everyone in my community can access healthy foods at affordable prices.”
  - “Everyone in my community has access to safe drinking water.”
  - “My community has safe outdoor places to walk and be physically active.”
  - “My community has decent and affordable housing available for everyone.”
  - “My community has public transportation, sidewalks for walking, and bike lanes available so people don’t always have to rely on cars.”
- Community connections (all use a 4-point scale for rating agreement with each question, Not at all to Completely):
  - “My community celebrates diversity.”
  - “My community hosts community-wide celebrations that represent all community members.”
  - “There is a high level of trust among members of my community.”
  - “Members of my community help and support each other, especially in times of need.”
  - “Members of my community know each other.”
  - “Members of my community are willing to work together to improve health.”
  - “Members of my community have a say in budgets and policy decisions.”

**Appendix Table A1. Ratings of individual well-being by socio-demographic characteristics**

|  | **Weighted %** | | | | **Mean** | **SD** |
| --- | --- | --- | --- | --- | --- | --- |
|  | **4 and below** | **5 to 6** | **7+** | **Missing** |  |  |
| **Sex** |  |  |  |  |  |  |
| Male | 13.4 | 23.4 | 63.0 | 0.2 | 7.04 | 1.87 |
| Female | 12.8 | 24.7 | 62.1 | 0.4 | 6.98 | 1.91 |
| **Race/Ethnicity*** |  |  |  |  |  |  |
| NH white | 12.2 | 21.4 | 66.1 | 0.3 | 7.16 | 1.80 |
| NH Black | 13.5 | 31.7 | 54.3 | 0.5 | 6.85 | 1.94 |
| Hispanic | 15.2 | 26.8 | 57.8 | 0.1 | 6.88 | 2.02 |
| NH Asian/PI | 12.1 | 26.9 | 60.2 | 0.8 | 6.96 | 1.78 |
| NH other | 19.1 | 29.0 | 51.9 | 0.0 | 6.63 | 2.10 |
| **Household Income*** |  |  |  |  |  |  |
| <$10k | 23.2 | 37.1 | 39.0 | 0.7 | 6.12 | 2.51 |
| $10k-25k | 25.9 | 25.9 | 47.9 | 0.2 | 6.27 | 2.31 |
| $25k-50k | 18.6 | 26.4 | 54.8 | 0.2 | 6.60 | 2.09 |
| $50k-75k | 12.5 | 26.4 | 61.0 | 0.1 | 6.95 | 1.87 |
| $75k-100k | 10.1 | 26.3 | 63.4 | 0.2 | 7.06 | 1.77 |
| $100k+ | 6.2 | 18.2 | 75.1 | 0.5 | 7.43 | 1.56 |
| **Age*** |  |  |  |  |  |  |
| 18-24 | 17.4 | 34.0 | 47.3 | 1.3 | 6.21 | 2.14 |
| 25-44 | 18.5 | 29.3 | 52.0 | 0.1 | 6.35 | 1.97 |
| 45-64 | 11.3 | 21.5 | 66.9 | 0.2 | 7.04 | 1.82 |
| 65+ | 5.1 | 14.7 | 80.0 | 0.2 | 7.67 | 1.60 |
| **Education*** |  |  |  |  |  |  |
| Less than HS | 21.8 | 30.1 | 47.7 | 0.4 | 6.37 | 2.34 |
| HS grade | 16.2 | 28.1 | 55.2 | 0.4 | 6.75 | 2.11 |
| Some college | 13.2 | 23.8 | 62.7 | 0.3 | 6.92 | 1.90 |
| College graduate | 8.7 | 20.9 | 70.3 | 0.1 | 7.20 | 1.62 |
| Postgraduate degree | 6.9 | 17.0 | 75.9 | 0.2 | 7.46 | 1.59 |
| **Urbanicity*** |  |  |  |  |  |  |
| Rural | 14.5 | 21.7 | 63.4 | 0.4 | 7.13 | 1.97 |
| Urban | 12.8 | 24.6 | 62.3 | 0.3 | 6.99 | 1.88 |

Table Notes: NH = Non-Hispanic; HS = high school. *Denotes that mean scores differ significantly among categories of each demographic characteristic, based on the Kruskal–Wallis equality-of-populations rank test (p < 0.01). Individual well-being measured with the Cantril Ladder: Assume the ladder is a way of picturing your life. On which step of this ladder would you say you personally feel you stand at this time? 0 is the worst possible life for you, 10 is the best possible life for you. Individual well-being responses grouped per methodology developed by Gallup to summarize results of the Cantril Scale.(51)

**Appendix Table A2.** **Correlations between elements of community well-being and individual well-being and overall community well-being**

| **Element of community well-being** | **Individual well-being** | **Community well-being** |
| --- | --- | --- |
| Overall community well-being | 0.291 | 1.00 |
| Conditions**:** |  |  |
| Health care | 0.183 | 0.369 |
| Healthy food | 0.181 | 0.376 |
| Safe drinking water | 0.146 | 0.277 |
| Outdoor spaces | 0.196 | 0.390 |
| Affordable housing | 0.157 | 0.226 |
| Transportation infrastructure | 0.053 | 0.119 |
| **Connections:** |  |  |
| Celebration of diversity | 0.079 | 0.204 |
| Community-wide events | 0.129 | 0.233 |
| Trust | 0.161 | 0.397 |
| Mutual support | 0.183 | 0.328 |
| Familiarity | 0.107 | 0.208 |
| Collaboration for health | 0.122 | 0.251 |
| Participation in decision-making | 0.109 | 0.242 |

**Appendix Table A3. Results of an ordered regression analysis of relationships between overall community well-being and demographics and specific elements of community well-being**

| **Factor** | **Coefficient (β)** | **(SE)** |
| --- | --- | --- |
| **Race/ethnicity (Reference group: NH white)** |  |  |
| NH Black | -0.191** | (0.0761) |
| Hispanic | -0.168** | (0.0735) |
| NH Asian/PI | 0.104 | (0.119) |
| NH other | -0.359** | (0.175) |
| **Gender (Reference group: male)** |  |  |
| Female | -0.0904* | (0.0528) |
| **HH income (Reference group: <$10k)** |  |  |
| $10k-25k | 0.0814 | (0.178) |
| $25k-50k | 0.270 | (0.164) |
| $50k-75k | 0.424*** | (0.165) |
| $75k-100k | 0.394** | (0.171) |
| $100k+ | 0.875*** | (0.164) |
| **Education (Reference group: <HS)** |  |  |
| HS graduate | 0.168 | (0.117) |
| Some college/associate’s degree | 0.251** | (0.117) |
| College graduate | 0.398*** | (0.124) |
| Master’s degree or above | 0.355*** | (0.129) |
| **Urbanicity (Reference group: rural)** |  |  |
| Urban | 0.380*** | (0.0793) |
| **Age (Reference group: < 25)** |  |  |
| 25-44 | -0.398*** | (0.129) |
| 45-64 | -0.425*** | (0.127) |
| 65+ | -0.147 | (0.130) |
| **Survey sample (Reference group: KnowledgePanel)** |  |  |
| ALP | -0.180*** | (0.0651) |
| **Elements of community well-being** |  |  |
| *Conditions:* |  |  |
| Health care | 0.491*** | (0.0664) |
| Healthy food | 0.574*** | (0.0664) |
| Safe drinking water | 0.140* | (0.0780) |
| Outdoor spaces | 0.874*** | (0.0745) |
| Affordable housing | 0.0466 | (0.0647) |
| Transportation infrastructure | -0.150** | (0.0583) |
| *Connections:* |  |  |
| Celebration of diversity | 0.0111 | (0.0648) |
| Community-wide events | -0.0894 | (0.0666) |
| Trust | 0.816*** | (0.0714) |
| Mutual support | 0.273*** | (0.0711) |
| Familiarity | 0.0405 | (0.0713) |
| Collaboration for health | 0.119 | (0.0741) |
| Participation in decision-making | 0.237*** | (0.0719) |

Table Notes: NH = Non-Hispanic; HS = high school. *** p<0.01, ** p<0.05, * p<0.1. For analysis of elements of well-being, we controlled for race/ethnicity, gender, income, education, urbanicity, survey sample, and age.

As a sensitivity check, we estimated regression models using the full ordinal scale for overall community well-being as the dependent variable (Table A3). Patterns of association in this model were generally consistent with those observed in the binary logistic model, indicating robustness of the findings to model specification. Slight differences emerged when comparing the logistic regression using the dichotomized community well-being measure with the ordered logistic regression that retained the full scale. In the ordered model, the association between collaboration for health and overall community well-being was no longer statistically significant, whereas the association between participation in decision-making and overall community well-being became statistically significant, indicating that the strength of these specific relationships varied somewhat depending on how the outcome was modeled.

**Table A4. Stepwise Binary Logistic Regression Models Predicting Favorable Community Well‑Being Ratings (Excellent/Very Good/Good vs. Fair/Poor) by Sociodemographics, Community Conditions, and Community Connections**

|  |  |  |  |  |  |  |  |
| --- | --- | --- | --- | --- | --- | --- | --- |
|  | | Model 1 | | Model 2 | | Full Model | |
|  | | SOCIODEMOGRAPHICS ONLY | | SOCIODEM + COMMUNITY CONDITIONS | | SOCIODEM + COMM CONDIT + COMM CONNECT | |
| VARIABLES | | q32exc-good | odds ratio | q32exc-good | odds ratio | q32exc-good | odds ratio |
| Race/ethnicity: NH Black | | -0.649*** | 0.523*** | -0.325*** | 0.722*** | -0.206* | 0.814* |
|  | | (0.0987) | (0.431 - 0.634) | (0.110) | (0.582 - 0.895) | (0.113) | (0.652 - 1.016) |
| Race/ethnicity: Hispanic | | -0.342*** | 0.710*** | -0.201* | 0.818* | -0.122 | 0.885 |
|  | | (0.101) | (0.582 - 0.866) | (0.110) | (0.659 - 1.016) | (0.113) | (0.709 - 1.106) |
| Race/ethnicity: NH Asian/PI | | 0.183 | 1.200 | 0.417* | 1.518* | 0.472** | 1.604** |
|  | | (0.208) | (0.798 - 1.806) | (0.226) | (0.975 - 2.363) | (0.234) | (1.014 - 2.537) |
| Race/ethnicity: NH other | | -0.787*** | 0.455*** | -0.560** | 0.571** | -0.510** | 0.600** |
|  | | (0.218) | (0.297 - 0.698) | (0.240) | (0.357 - 0.914) | (0.251) | (0.367 - 0.982) |
| Sex: Female | | -0.114 | 0.893 | -0.0188 | 0.981 | -0.0245 | 0.976 |
|  | | (0.0751) | (0.770 - 1.034) | (0.0820) | (0.836 - 1.152) | (0.0844) | (0.827 - 1.151) |
| Income: $10k-<25k | | 0.213 | 1.237 | 0.145 | 1.156 | 0.130 | 1.139 |
|  | | (0.183) | (0.864 - 1.772) | (0.203) | (0.777 - 1.719) | (0.210) | (0.755 - 1.719) |
| Income: $25k-<50k | | 0.533*** | 1.705*** | 0.361* | 1.435* | 0.337* | 1.401* |
|  | | (0.170) | (1.222 - 2.379) | (0.188) | (0.992 - 2.076) | (0.196) | (0.955 - 2.057) |
| Income: $50k-<75k | | 0.922*** | 2.515*** | 0.668*** | 1.950*** | 0.607*** | 1.835*** |
|  | | (0.176) | (1.783 - 3.548) | (0.194) | (1.334 - 2.851) | (0.201) | (1.238 - 2.719) |
| Income: $75k-<100k | | 0.960*** | 2.611*** | 0.672*** | 1.958*** | 0.591*** | 1.806*** |
|  | | (0.186) | (1.815 - 3.756) | (0.205) | (1.309 - 2.928) | (0.213) | (1.189 - 2.744) |
| Income: $100k+ | | 1.547*** | 4.696*** | 1.154*** | 3.170*** | 1.085*** | 2.960*** |
|  | | (0.177) | (3.317 - 6.648) | (0.197) | (2.156 - 4.660) | (0.204) | (1.985 - 4.413) |
| Education: HS graduate | | 0.151 | 1.163 | 0.0707 | 1.073 | 0.0888 | 1.093 |
|  | | (0.140) | (0.884 - 1.529) | (0.155) | (0.792 - 1.454) | (0.159) | (0.801 - 1.491) |
| Education: Some college or Associate degree | | 0.269* | 1.308* | 0.134 | 1.143 | 0.142 | 1.153 |
|  | | (0.142) | (0.990 - 1.729) | (0.158) | (0.839 - 1.558) | (0.162) | (0.840 - 1.582) |
| Education: Bachelor’s degree | | 0.396** | 1.485** | 0.137 | 1.147 | 0.183 | 1.200 |
|  | | (0.160) | (1.085 - 2.032) | (0.177) | (0.810 - 1.622) | (0.181) | (0.842 - 1.711) |
| Education: Master’s degree or above | | 0.140 | 1.150 | -0.0936 | 0.911 | -0.0580 | 0.944 |
|  | | (0.168) | (0.828 - 1.598) | (0.184) | (0.635 - 1.307) | (0.189) | (0.652 - 1.366) |
| Urbanicity: Urban | | 0.298*** | 1.347*** | 0.328*** | 1.389*** | 0.358*** | 1.431*** |
|  | | (0.105) | (1.096 - 1.655) | (0.115) | (1.109 - 1.738) | (0.120) | (1.131 - 1.809) |
| Age: 25-44 | | -0.549*** | 0.578*** | -0.627*** | 0.534*** | -0.592*** | 0.553*** |
|  | | (0.178) | (0.408 - 0.819) | (0.201) | (0.360 - 0.792) | (0.206) | (0.370 - 0.828) |
| Age: 45-64 | | -0.453** | 0.636** | -0.673*** | 0.510*** | -0.625*** | 0.535*** |
|  | | (0.177) | (0.450 - 0.899) | (0.200) | (0.345 - 0.755) | (0.205) | (0.358 - 0.800) |
| Age: 65+ | | 0.211 | 1.235 | -0.155 | 0.857 | -0.103 | 0.903 |
|  | | (0.187) | (0.857 - 1.781) | (0.210) | (0.567 - 1.294) | (0.216) | (0.591 - 1.378) |
| Survey sample: ALP | | -0.0880 | 0.916 | -0.128 | 0.880 | -0.183* | 0.833* |
|  | | (0.0957) | (0.759 - 1.105) | (0.103) | (0.719 - 1.076) | (0.105) | (0.678 - 1.024) |
| Everyone has access to needed health care, services | |  |  | 0.479*** | 1.614*** | 0.332*** | 1.394*** |
|  | |  |  | (0.0977) | (1.333 - 1.955) | (0.101) | (1.144 - 1.698) |
| Everyone can access healthy foods at affordable prices | |  |  | 0.633*** | 1.883*** | 0.513*** | 1.671*** |
|  | |  |  | (0.112) | (1.512 - 2.345) | (0.116) | (1.332 - 2.096) |
| Everyone has access to safe drinking water | |  |  | 0.265*** | 1.304*** | 0.211** | 1.235** |
|  | |  |  | (0.0969) | (1.078 - 1.577) | (0.1000) | (1.016 - 1.503) |
| Safe outdoor places to walk and be physically active | |  |  | 1.079*** | 2.941*** | 0.916*** | 2.498*** |
|  | |  |  | (0.0955) | (2.439 - 3.547) | (0.0984) | (2.060 - 3.030) |
| Decent and affordable housing available for everyone | |  |  | 0.343*** | 1.410*** | 0.116 | 1.123 |
|  | |  |  | (0.114) | (1.127 - 1.764) | (0.120) | (0.887 - 1.421) |
| Public transportation, sidewalks, bike lanes available | |  |  | -0.166* | 0.847* | -0.266*** | 0.766*** |
|  | |  |  | (0.0899) | (0.711 - 1.011) | (0.0963) | (0.635 - 0.926) |
| My community celebrates diversity | |  |  |  |  | 0.102 | 1.107 |
|  | |  |  |  |  | (0.110) | (0.892 - 1.375) |
| Community-wide celebrations that represents all members | |  |  |  |  | 0.0122 | 1.012 |
|  | |  |  |  |  | (0.112) | (0.813 - 1.261) |
| High level of trust among members of my community | |  |  |  |  | 0.733*** | 2.081*** |
|  | |  |  |  |  | (0.135) | (1.597 - 2.711) |
| Members help, support each other in times of need | |  |  |  |  | 0.551*** | 1.735*** |
|  | |  |  |  |  | (0.120) | (1.370 - 2.197) |
| Members of my community know each other | |  |  |  |  | -0.173 | 0.841 |
|  | |  |  |  |  | (0.124) | (0.660 - 1.072) |
| Willing to work together to improve health | |  |  |  |  | 0.275** | 1.316** |
|  | |  |  |  |  | (0.138) | (1.004 - 1.727) |
| Have a say in budgets and policy decisions | |  |  |  |  | 0.0683 | 1.071 |
|  | |  |  |  |  | (0.141) | (0.812 - 1.411) |

Table Notes: NH = Non-Hispanic; HS = high school. *** p<0.01, ** p<0.05, * p<0.1.
